# Lipophilic dye-compatible brain clearing technique allowing correlative magnetic resonance/high-resolution fluorescence imaging in rat models of glioblastoma

**DOI:** 10.1038/s41598-020-75137-y

**Published:** 2020-10-21

**Authors:** Marco Peviani, Giorgia Spano, Antonella Pagani, Gianluca Brugnara, Cesare Covino, Rossella Galli, Alessandra Biffi, Letterio S. Politi

**Affiliations:** 1 Gene Therapy Program, Dana-Farber/Boston Children’s Centre for Cancer and Blood Disorders, Boston, MA USA; 2 Harvard Medical School, Boston, MA USA; 3 Neuroradiology Unit and CERMAC, Vita-Salute San Raffaele University and IRCCS San Raffaele Scientific Institute, Milan, Italy; 4 Advanced Light and Electron Microscopy Imaging Centre, IRCCS San Raffaele Scientific Institute, Milan, Italy; 5 Neural Stem Cell Biology Unit, Division of Neuroscience, IRCCS San Raffaele Scientific Institute, Milan, Italy; 6 Department of Neuroradiology, Heidelberg University Hospital, Heidelberg, Germany; 7 Section for Computational Neuroimaging, Department of Neuroradiology, Heidelberg University Hospital, Heidelberg, Germany; 8 Neuroimaging Research, Hematology/Oncology Division and Radiology Dept., Boston Children’s Hospital, 300 Longwood Ave, Boston, MA 02215 USA; 9 Advanced MRI Center and Radiology Department, University of Massachusetts Medical School, Worcester, MA USA; 10 Neuroradiology Department, Humanitas University and Humanitas Clinical and Research Hospital IRCCS, Milan, Italy; 11 Department of Biology and Biotechnology “L. Spallanzani”, University of Pavia, Via Ferrata 9, 27100 Pavia, Italy

**Keywords:** Cancer imaging, Cancer models, Magnetic resonance imaging, 3-D reconstruction

## Abstract

In this work we optimized a novel approach for combining in vivo MRI and ex vivo high-resolution fluorescence microscopy that involves: (i) a method for slicing rat brain tissue into sections with the same thickness and spatial orientation as in in vivo MRI, to better correlate in vivo MRI analyses with ex-vivo imaging via scanning confocal microscope and (ii) an improved clearing protocol compatible with lipophilic dyes that highlight the neurovascular network, to obtain high tissue transparency while preserving tissue staining and morphology with no significant tissue shrinkage or expansion. We applied this methodology in two rat models of glioblastoma (GBM; U87 human glioma cells and patient-derived human glioblastoma cancer stem cells) to demonstrate how vital the information retrieved from the correlation between MRI and confocal images is and to highlight how the increased invasiveness of xenografts derived from cancer stem cells may not be clearly detected by standard in vivo MRI approaches. The protocol studied in this work could be implemented in pre-clinical GBM research to further the development and validation of more predictive and translatable MR imaging protocols that can be used as critical diagnostic and prognostic tools. The development of this protocol is part of the quest for more efficacious treatment approaches for this devastating and still uncurable disease. In particular, this approach could be instrumental in validating novel MRI-based techniques to assess cellular infiltration beyond the macroscopic tumor margins and to quantify neo-angiogenesis.

## Introduction

Glioblastoma (GBM) is the most common primary brain tumor and the most aggressive brain cancer. It is characterized by a fast growth rate and high invasiveness of the cancer cells^1^. The prognosis is poor, with median survival of about 15 months after diagnosis^2,3^. One of the major hurdles in GBM therapy is the presence of infiltrating cancer cells that have moved away from the main tumor mass and are therefore difficult to remove during surgery^4^. Validating magnetic resonance (MR)-based techniques for the identification of infiltrating tumor cells beyond the macroscopic margins of the tumoral mass may be a crucial step toward improving patient survival by guiding neurosurgeons in performing supramarginal tumor resections^5,6^. Furthermore, accumulating evidence suggests that much of cancer therapy resistance and a tumor’s ability to regenerate after the bulk of the tumor mass has been treated may depend on a small population of cells known as cancer stem-like cells (CSCs)^7,8^. The identification of CSCs and the development of therapeutic approaches capable of targeting them before they spread and contribute to tumor recurrence is critical to improving the success of cancer therapy.

Recent advances in Magnetic Resonance Imaging (MRI) have made it possible to quantify water diffusion between the intracellular and extracellular compartments, potentially making it possible to distinguish edema from cell infiltration. Recent advances have also made it possible to quantify tumor perfusion and permeability of neo-angiogenetic vasculature^9–13^. However, there is still urgent need to develop and validate new MRI-based approaches that are capable of providing detailed information on critical markers of prognosis and response to treatment, such as tumor volume, cellularity, vascularization of the tumor mass, and spreading of cancer cells within the brain parenchyma.

Tumor-induced angiogenesis is a key mechanism that drives tumor growth. New preclinical techniques for visualizing and studying tumor/blood vessel networks in GBM could provide new insights into tumor biology and help identify and validate innovative therapeutic approaches^14,15^. One of the approaches for visualizing the vascular network is called “vessel painting”^16–18^ and it will be referred as “vessel labeling” in this manuscript. The protocol relies on the intracardiac perfusion with DiI, a lipophilic carbocyanine dye that passively diffuses into the lipid membranes of the endothelial cells. This allows the study of the vasculature by fluorescence microscopy without the need for immunohistochemistry. Recent advances in tissue-clearing and microscopy techniques have dramatically improved the possibilities for investigating the brain architecture in preclinical models, shedding light on the morphological features of tumor mass and the anatomical distribution of tumor cells – including their interactions with the vasculature network^19–21^. Several methodologies have been proposed for brain tissue clearing and each one presents its own advantages and drawbacks. Among the latter, variability in the performance of clearing the whole adult mouse brain, modification of tissue dimensions (expansion versus shrinkage), and compatibility with fluorescent proteins and/or with lipophilic dyes are to be considered. Refer to^22,23^ for a comprehensive list of features for the different clearing protocols.

In this work we present an approach that correlates ex-vivo high-resolution fluorescence microscopy images that visualize tumor cell invasion and brain vasculature with in vivo MRI. We apply a modified DiI-compatible tissue clearing protocol to simultaneously guarantee high tissue transparency, compatibility with a lipophilic-dye based vessel labeling protocol, preservation of fluorescent proteins and, most importantly, no induction of significant tissue expansion or shrinkage. We coupled this approach with a newly-developed method for slicing rat brain tissue into sections with the same thickness and spatial orientation adopted for in vivo MRI. This combined approach allows for a whole-slice, high-resolution investigation of tumor cell biodistribution and visualizes tumor cell interaction with the brain vasculature. This visualization is correlated side-by-side with the anatomical readouts obtained in vivo through MRI. Taken together, this represents a new method that can be employed to validate novel advanced MRI-based techniques, and furthermore, highlights the limitation of standard morphological MRI imaging sequences such as T2-weighted and post-contrast T1-weighted imaging.

## Results

### Tumor growth, monitored by MRI, correlates with histological readouts and cancer cell type

**Figure 1:**
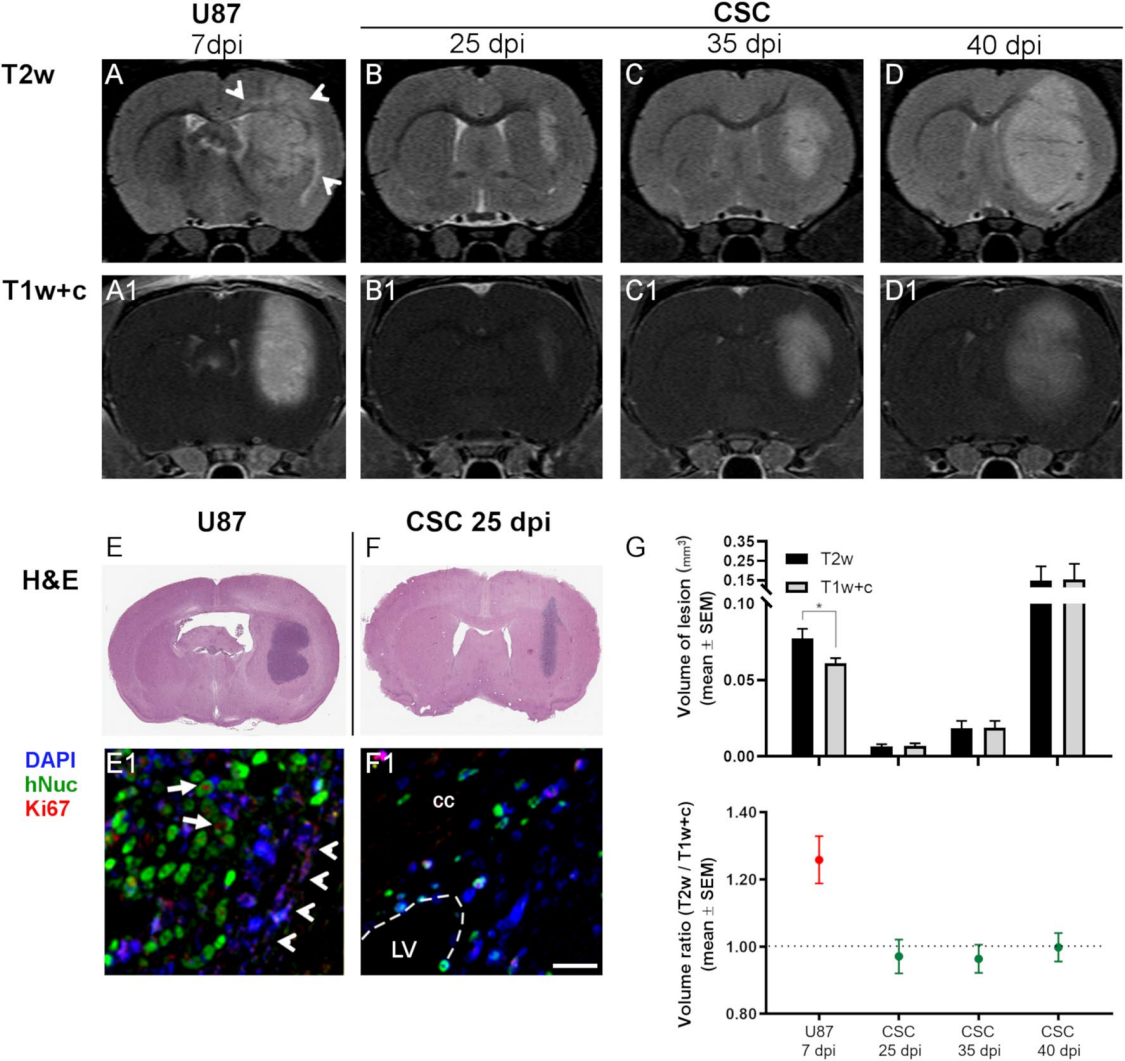
Differences between intracranial xenografts from U87 or Cancer Stem Cell (CSC) lines highlighted by MRI. Representative T2-weighted (T2w) (**A**–**D**) and post-contrast T1-weighted (T1w + c) (**A1**–**D1**) brain coronal sections of U87 or CSC xenografts in Rnu rats. A lower growth rate and reduced contrast uptake is observed in tumors obtained from CSCs, as compared to U87. In contrast to CSCs, in U87 xenografts the area of hyperintensity detected on T2w images (also involving the cortex and the majority of the homolateral part of the corpus callosum) is larger than that identified on T1w + c. Pathology demonstrated the absence of tumor infiltration at this level, and therefore this corresponds to perilesional edema. (**E**, **F**) H&E staining of brain slices from representative rats transplanted with U87 or CSCs (7 or 25 dpi, respectively), showing higher growth rate of U87. (**E1**, **F1**) High magnification laser scanning confocal microphotographs of human nuclei (green signal) and DAPI stain (blue) on U87 or CSC-tumor bearing representative rats, highlighting higher invasiveness of CSC xenografts: CSC derived cells extend to the corpus callosum (cc) and to the ependymal layer of the lateral ventricle (LV); in contrast, U87 cells remain confined within the tumor boundary, highlighted by arrowheads (scale bar = 25 μm). (**G**) Graphs showing the volume of lesion measured on T2w or T1w + c images (upper panel) and the ratio between the volume of the lesion measured on T2w versus T1w after contrast (lower panel). In U87 xenografts the volume of hyperintensity detected on T2w images is significantly larger than that identified on T1w + c (*p < 0.05; Wilcoxon’s test). In CSCs xenografts the volume of the lesion highlighted with MRI is similar between T2w and T1w + c images. The ratio for CSCs xenografts is slightly below 1.0 at 25 and 35 dpi (0.97 ± 0.05 and 0.96 ± 0.04, respectively) and equals 0.99 ± 0.04 at 40 dpi; in contrast, for U87 xenografts a significant increase of volume ratio is detected (1.26 ± 0.07; p < 0.01; one-way ANOVA).

Firstly, we generated a preclinical GBM model by transplanting human cancer cells (U87 and patient derived CSCs) into immunocompromised Rowett nude (Rnu) rats. While the U87 cancer cell line has been widely used in GBM preclinical in vivo investigations, the tumors generated with this cell line fail to recapitulate some critical aspects of GBM pathology, such as high invasiveness and spreading throughout the brain parenchyma^24^. For this reason, we also chose to explore the use of patient-derived cancer stem cells (CSCs) that are known to give rise to infiltrative xenografts^25^. U87 or CSCs (2.5 × 10^5^ or 5 × 10^5^ cells, respectively) were injected into the right striatum of Rnu rats (n ≥ 4 animals/cell type/time-point). The progression of the tumor in animals was monitored over-time, at 7, 25, 35, and 40 days post-injection (dpi), through MRI (T2w and T1w post-contrast). In the case of U87-derived GBM, animals had to be euthanized at 7 dpi due to the rapid deterioration of their condition, consistent with the strikingly rapid growth rate of the U87-derived tumors (Fig. 1A). In detail, in U87 xenografts the area of hyperintensity detected on T2w images (also involving the cortex and the majority of the ipsilateral part of the corpus callosum—arrowheads in Fig. 1A–A1) is larger than that identified on T1w + c. Pathological analysis demonstrated the absence of tumor infiltration in the non-enhancing T2 hyperintense area, in particular in the corpus callosum, and therefore this area of T2 hyperintensity without contrast enhancement corresponds to perilesional edema, as confirmed by H&E staining (Fig. 1E). On the other hand, the growth rate of the CSC-derived xenograft was slower (as evidenced by MRI, Fig. 1B–D), requiring up to 40 days to determine tissue alterations (as evidenced by T2w and T1w + c imaging in Fig. 1D–D1, respectively) and deterioration of animal’s conditions comparable to that observed in U87 xenografts at 7dpi. Overall, the tumor volume calculated on post-contrast T1w images (T1w + c) in CSC-derived xenografts was significantly smaller than that measured in the U87 xenografts until 35 dpi (Fig. 1B1,C1 and quantification in Fig. 1G). Moreover, in the case of CSC-derived xenografts, the area of contrast enhancement shown on T1w + c images (Fig. 1B1–D1) was not statistically different from the hyperintensity on T2w images (Fig. 1B–D and quantification in Fig. 1G), suggesting that in this GBM model the T2 hyperintensity mainly represents tumor invasion and not edema. By tracking the xenografts-derived cells through immunohistochemistry for human nuclei (hNuc), we confirmed that U87 cells (Figs. 1E1, S1A) remain confined within the macroscopic tumor boundaries (arrowheads in Fig. 1E1) and are highly proliferative, as evidenced by the co-localization of hNuc with Ki67 proliferation marker (arrows in Figs. 1E1 and S1A–C). On the other hand, CSC-derived xenografts displayed a reduced cellularity as compared to U87-derived tumors (Fig. 1F), which is in line with the MRI data. Immunohistochemistry for hNuc highlighted human cells in CSC-transplanted rats localized in regions distant from the injection site, such as the corpus callosum (cc) and the ependymal cell layer of lateral ventricle (LV) homolateral to the injection site (Figs. 1F1, S1D–F). This confirms the infiltrating behaviour of the CSC model.

### Co-locatization of MRI and pathology slices

**Figure 2:**
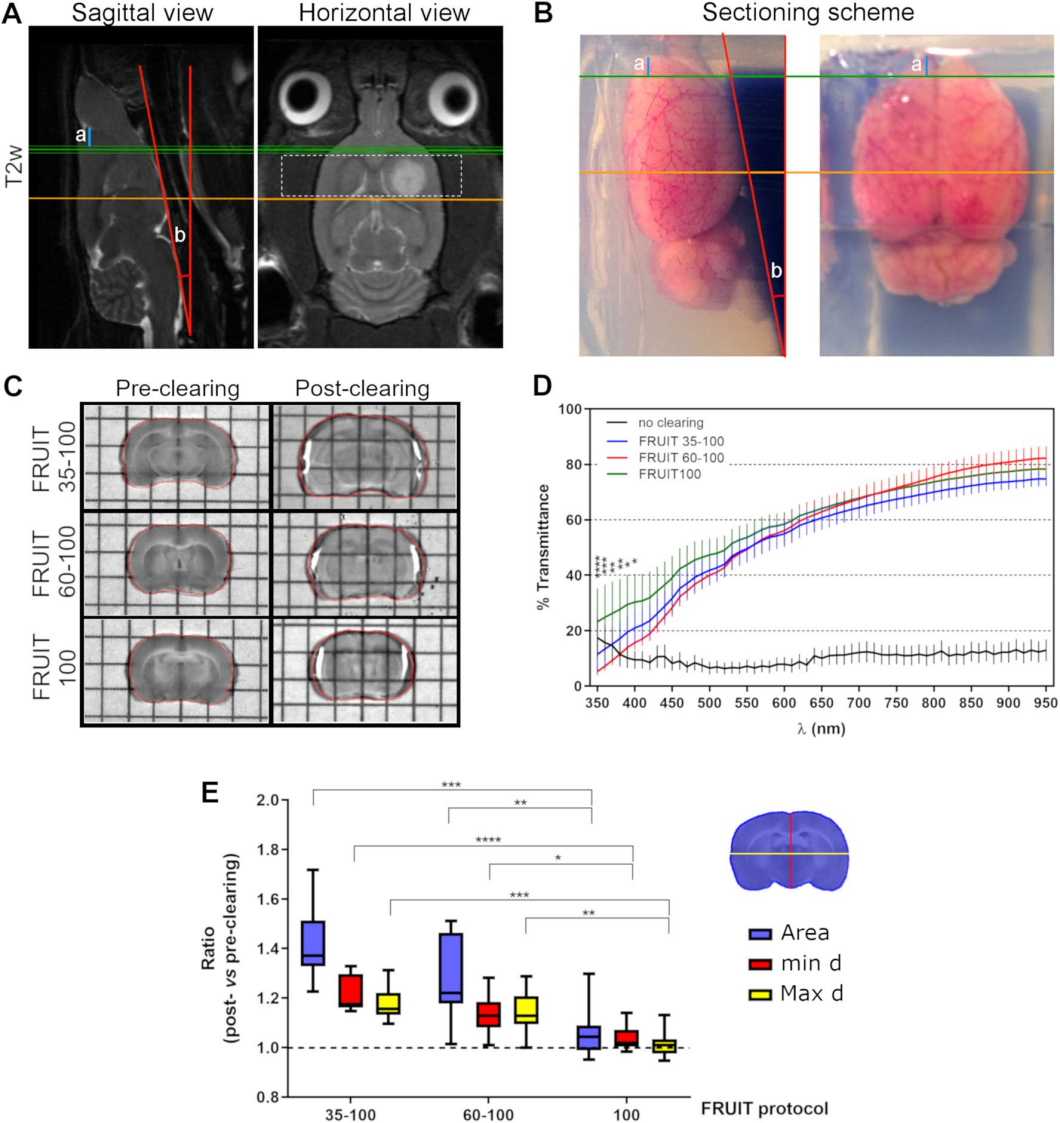
Outline of tissue processing and clearing protocols. (**A**) Representative T2-weighted sagittal and horizontal brain sections of a CSC xenograft at 35 dpi, showing the reference coordinates used to guide the ex-vivo tissue sectioning: red lines identify the orientation (angle b) versus the plane perpendicular to the position of the MRI images; green and orange lines identify respectively the starting and terminal points of the volume sampled by high resolution MRI; blue segment (a) identify the distance of the green line from the frontal pole. (**B**) PFA-fized brain tissue, collected after sacrifice, is embedded in an agarose mold following the orientation and reference distances identified in A, to allow sectioning in the coronal plane. (**C**) Representative pictures of brain slices processed with different clearing protocols. Tissue dimension and transparency are shown before (Pre) and after (Post) the clearing procedure. (**D**) Quantification of the pre- and post-clearing transmittance of the tissue slices shown in (**C**). All the three tested methods display a similarly high level of transparency (transmittance ≥ 50%) for wavelengths in the 550–950 nm range. For wavelengths in the 350–500 nm range, FRUIT 100 protocol determined a slight but significantly higher transmittance, as compared to the other clearing methods (*p < 0.05; **p < 0.01; ***p < 0.001; ****p < 0.00001; 1-way ANOVA followed by Tukey’s post-hoc test; n = 6 slices/group, 2 slices/animal). (**E**) Quantitative analysis of tissue expansion after application of different clearing protocols shown in C: the ratio of slice surface (area), minimal (min d) and maximal (max d) diameter before versus after clearing were evaluated using Fiji v.1.52p; https://imagej.net/Fiji. Protocols FRUIT 35–100 and 60–100 determined significant tissue expansion, whereas with protocol FRUIT 100 the tissue dimensions were not significantly affected. *p < 0.05; Kruskal–Wallis.

A critical aspect for understanding the performance of MRI protocols is to confirm the relevant features highlighted by in vivo MRI through an ex vivo histological examination of the same tissue. To refine the precision of this comparison, we developed a method for tissue sectioning that takes into account the spatial orientation of acquired images in respect to anatomical landmarks during MRI image acquisition (Fig. 2A). In detail, once the field of view of the MRI sequences was identified (dashed square in Fig. 2A) on reference images obtained on the three planes, we measured the distance (segment “a”, Fig. 2A) between the first slice of acquisition (green line, Fig. 2A) and the frontal pole, and the angle (b, Fig. 2A) between the ventral brain and the plane perpendicular to the acquired images. Then, after MRI, the animal was euthanized following the vessel labeling protocol and its brain was dissected and post-fixed. Finally, the tissue was cut into slices with the same thickness and orientation applied for the MRI images. In order to maintain the same spatial orientation for sectioning that was used in the MRI setting, the brain was included in 1% agar and oriented relative to the blade (Fig. 2B), according to the references (segment “a” and angle “b”) measured on the MRI images.

To verify the accuracy of this procedure, we injected paramagnetic superoxide-ion nanoparticles (SPIONs, 10 mg/ml iron; 5 μl/injection site) monolaterally, in the striatum or in the corpus callosum of Wistar rats (tot. n = 2) where no tumor had been transplanted. A sham injection was performed on the contralateral side, to control for possible unspecific signal coming from the procedure, such as bleeding or local inflammatory response surrounding the needle tract at the injection site. Rats were then imaged in vivo through MRI (T2w) 3 dpi, to highlight the localization of the SPIONs at the injection sites (hypointense signal in Fig. S2A,D). Then, the brain was dissected, processed and sectioned as described above. The anatomical structures, such as hippocampus (hip), lateral ventricles (LV), corpus callosum (cc) and the localization of the injection site and SPIONs biodistribution, as evidenced in vivo by MRI (Fig. S2A,D), were correctly sampled ex vivo upon brain slicing (Fig. S2B, E) and after histology (Fig. S2C,F). In the latter case, Prussian Blue staining was performed to highlight the iron of the contrast media, i.e. SPIONs localization (arrows in Fig. S2C,F) and Nuclear Fast-Red staining was performed on the same slices to highlight the anatomical structures.

### Implementation of an optimized DiI-compatible tissue clearing protocol that allows for high-resolution fluorescent microscope imaging of thick tissue sections

Vessel labeling through intracardiac perfusion with a solution containing DiI has been used to trace the neurovasculature by fluorescent microscopy in mice^16,17^. We used this technique to highlight the brain vasculature in the context of the Rnu GBM rat model.

In order to make a volumetric 3D reconstruction of the neurovasculature and the tumor cell distribution in each of the tissue sections corresponding to the MRI sampling, we used tissue clearing. Among the available techniques for tissue clearing, we adopted a protocol named FRUIT that is based on incubating the tissue in detergent-free solutions consisting of increasing concentrations of fructose and urea^26^. We utilized this protocol to achieve good tissue transparency while simultaneously preserving the vessel staining obtained with lipophilic dyes, such as DiI. However, one of the main limitations of this protocol—and of others based on the same principle, such as SeeDB^27^—is that the dimensions of the original tissue are not preserved. In detail, by applying the standard FRUIT protocol (subsequent incubations in fructose gradients ranging from 35 to 100%—see “Materials and methods” section—hereafter called FRUIT 35–100) we observed a significant expansion of the cleared section, as compared to its initial dimensions (Fig. 2C and quantification in Fig. 2E). As already described, the initial incubation steps in solutions containing lower fructose concentration are responsible for inducing the highest tissue expansion^26^. For this reason, we tested variants to the original FRUIT protocol that rely on higher concentration of fructose solution during the initial incubation steps. Variations include FRUIT 60–100, where an initial incubation in fructose 60% for 8 h was followed by 12 h-incubation in 80% (wt/vol) FRUIT and then a 24 h-incubation in 100% (wt/vol) FRUIT, and FRUIT 100, which involves a direct transfer of the tissue slice to a solution of fructose 100% for 24 h at 37 °C. The latter protocol showed the best results, since it facilitated optimal clearing (Fig. 2C and quantification of tissue transmittance in Fig. 2D) while causing negligible changes to the original dimensions of the tissue slice before clearing (Fig. 2C and quantification in Fig. 2D). Therefore, it was the only protocol employed for the following experiments.

### High-resolution imaging of cleared brain sections highlights the underestimation of tumor spread by standard MRI techniques

**Figure 3:**
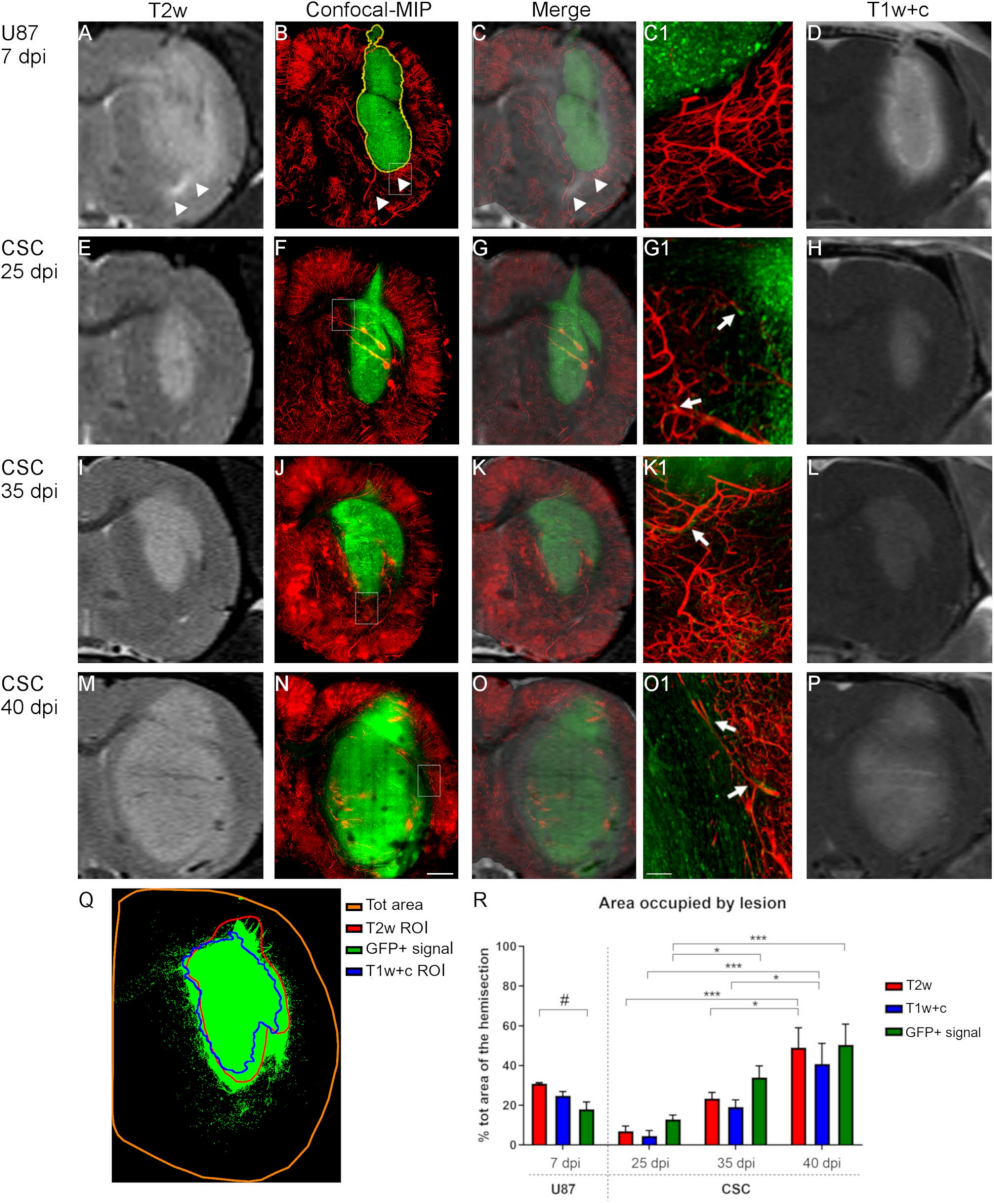
MRI/confocal analysis of tumor growth in U87- and CSC-derived GBM xenografts. Representative brain coronal sections of U87 or CSC xenografts imaged in vivo by T2-weighted (T2w) or post-contrast T1 weighted (T1w + c) images, and then ex-vivo by laser scanning confocal microscopy after vessel labeling, sectioning and tissue clearing (MIP = maximal intensity projection of 0.4 mm z-stack; vessels are stained in red with DiI; tumor cells expressing GFP reporter gene are colored in green). Superimposition of the confocal images on the respective T2w scans highlights for U87 xenografts a sharp delimitation of the tumor mass with no cells growing beyond the tumor boundary (yellow line in **B**). Moreover, a hyper-intense signal is detected on T2w (arrowheads in **A**–**C**) which is not associated to tumor cells. In the case of CSC xenografts, the tumor boundary is less evident and many cells diffuse throughout the brain parenchyma. Insets (**C1**, **G1**, **K1** and **O1**) show high magnification of the regions highlighted by dotted box in (**B**, **F**, **J** and **N**), respectively. Arrows in (**G1**, **K1** and **O1**) highlight GFP + tumor cells spread throughout the brain parenchyma, closely juxtaposed to the neurovasculature stained in red with DiI. (**D**, **H**, **L**, **P**) T1w + c images of the same samples shown in (**A**, **E**, **I**, **M**). Scale bar in (**N**) applies to (**A**–**O**) = 1 mm; scale bar in (**O1**) applies to (**C1**, **G1** and **K1**) = 100 µm. (**Q**) Representative picture showing the super-imposition of the hyper-intense signal identified on the T2w (red line) or T1w + c (blue line) scans and the GFP + signal detected by confocal microscopy (green signal) after tissue clearing. (**R**) Quantitative comparison of the precentage of the total hemi-section area occupied by the signal identified on the T2w, T1w + c or confocal scans, in animals receiving U87 or CSC xenografts (^#^p < 0.05; 1-way ANOVA followed by Tukey’s post-hoc test; *p < 0.05; ***p < 0.01; 2-way ANOVA followed by Tukey’s post-hoc test; n ≥ 4 samples/group). Co-registration of the confocal images with the corresponding digital slice obtained by MRI was done using GIMP (GNU Image Manipulation Program v.2.10.8; https://gimp.org). Image segmentation and quantification of the area occupied by GFP + cells or by the hyperintense signal highlighted with T2w or T1w + c was done using Fiji v.1.52p; https://imagej.net/Fiji.

**Figure 4:**
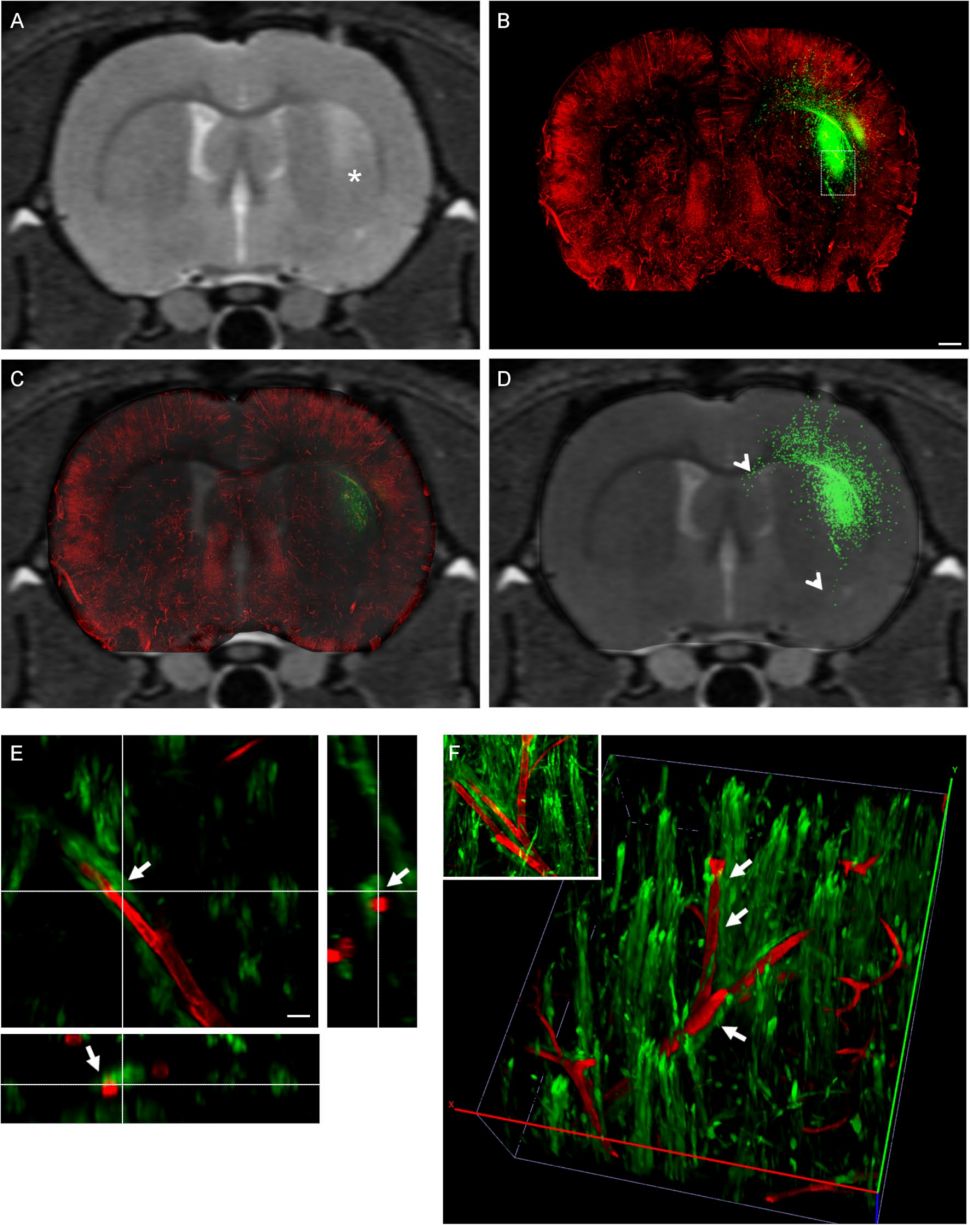
Confocal microscopy highlights spreading of CSCs into brain parenchyma beyond macroscopic tumoral margins. Representative in vivo brain coronal T2w images of CSC xenograft 25 days post-injection (**A**), and the corresponding ex-vivo laser scanning confocal microscopy image after vessel labeling, sectioning and tissue clearing (**B**); a full reconstruction of the entire slice was performed in this case. The region acquired is close to the injection site of the human GFP-labelled CSCs. (**C**) Superimposition of the confocal image on the respective T2w scan highlights the diffusion of many GFP + cells (arrowheads in **D**) beyond the area of hyperintense signal on the T2w image (asterisk in **A**). (**E**, **F**) High resolution confocal z-stack, 0.5um step size (**E**) and corresponding 3D reconstruction (**F**) of the region highlighted by the dashed box in B. Inset in F is a maximal intensity projection of the z-stack acquisition shown in (**E**). Arrows in (**E**, **F**) highlight the close juxtaposition of GFP + cells to brain vessels. Scale bar in (**B**) applies to (**A**–**D**) = 1 mm; scale bar in (**E**) = 100 µm.

**Figure 5:**
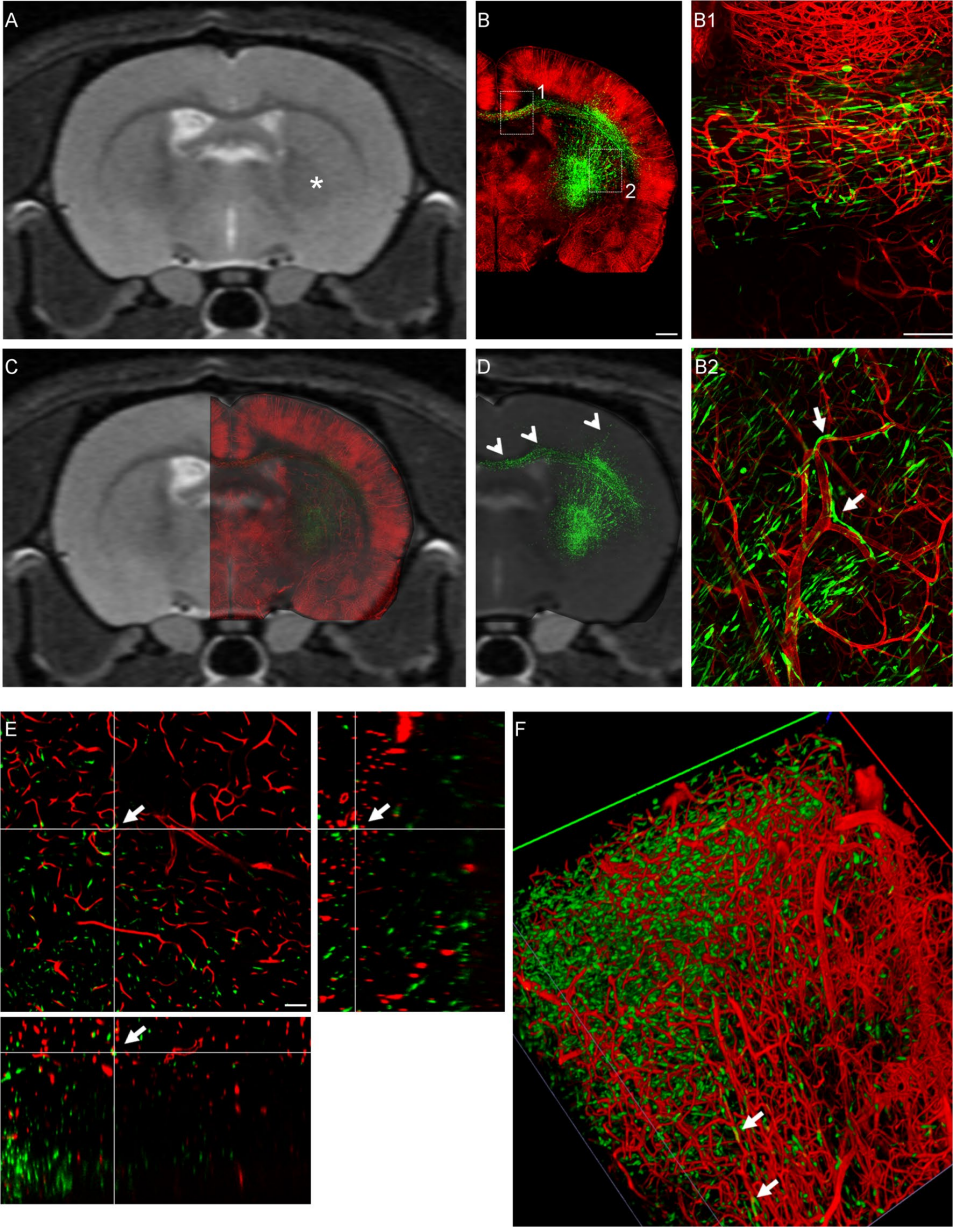
T2w MRI scans fail to identify the presence of CSC-derived tumor cells in brain regions distal from the injection site, as evidenced by confocal and 2-photon microscopy. Representative brain coronal section of CSC xenograft 25 days post-injection, imaged in vivo by MRI T2 weighted (T2w) scans (**A**), and then ex-vivo by laser scanning confocal microscopy after vessel labeling, sectioning and tissue clearing (**B**). The region acquired is 1.5 mm caudal to the injection site of the human GFP-labelled CSCs. (**C**) Superimposition of the confocal image on the respective T2w scan highlights widespread diffusion of many GFP + cells (arrowheads in **D**) throughout the striatum, cortex and corpus callosum. Despite many GFP + cells are detected in the brain in the area homolateral to the injection site, in this case no hyperintense signal is detected by the T2w scan (asterisk in **A**). (**B1**, **B2**) High resolution confocal maximal intensity projection of the regions highlighted by the dashed boxes in B. (**E**) High resolution 750 μm-thick z-stack (0.5 μm step size) acquired by 2-photon microscopy. (**F**) 3D reconstruction of the region shown in (**E**). Arrows in B2, (**E**, **F**) highlight the close juxtaposition of GFP + cells to brain vessels. Scale bar in (**B**) applies to (**A**–**D**) = 1 mm; scale bar in (**E**) = 100 µm.

**Figure 6:**
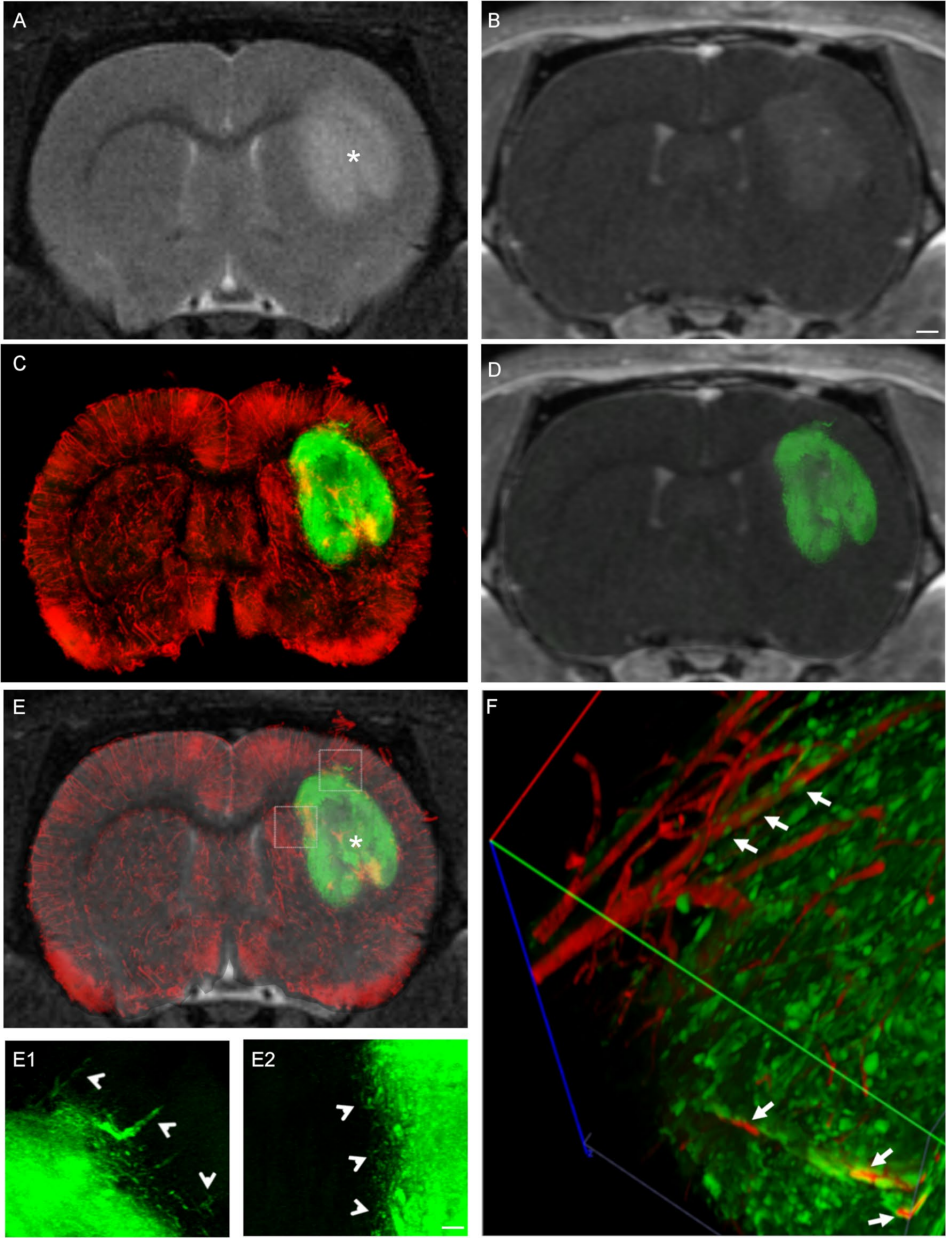
Two-photon microscopy of the full tissue thickness confirms the spreading of CSC-derived tumor cells beyond the macroscopic tumoral margins identified with T2w and T1w + c MRI. Representative brain coronal section of CSC xenograft 35 days post-injection, imaged in vivo by MRI T2 weighted (T2w) scans (**A**), T1w post-contrast (T1w + c) imaging (**B**), and then ex-vivo by 2-photon microscopy after vessel labeling, sectioning and tissue clearing (**C**). The region acquired is close to the injection site of the human GFP-labelled CSCs (asterisk in **A**, **E**). Superimposition of the 2-photon acquisition on the respective T1w + c (**D**, GFP + signal only) and on T2w (**E**, DiI-stained vessels and GFP + cancer cells) scans highlights diffusion of the GFP + cells throughout the striatum and cortex. (**E1**, **E2**) High resolution maximal intensity projection of the regions highlighted by the dashed boxes in (**E**). Arrowheads highlight the GFP + cells detected beyond the macroscopic tumor margins. (**F**) 3D reconstruction of a 2-photon z-stack. Arrows in (**F**) highlight GFP + cells juxtaposed to brain vessels. Scale bar in (**B**) applies to (**A**–**E**) = 1 mm; scale bar in (**E1**, **2**) = 100 µm.

The optimized tissue clearing protocol (FRUIT100), coupled with Dil vessel labeling and confocal and 2-photon microscopy, was finally applied to an ex vivo study of the extent of correlation between MRI analyses and the biodistribution of tumor cells in Rnu GBM models at different stages post cell implant. In order to be able to trace the cancer cells by fluorescent microscopy, U87 and CSC lines were first transduced with a lentiviral vector expressing GFP reporter gene under a ubiquitous promoter^25^. We first utilized flow cytometry to confirm high and homogeneous levels of GFP expression in both U87 and CSC cell cultures in order to better guarantee detection of these cells after transplantation in Rnu rats. U87 or CSCs (2.5 × 10^5^ or 5 × 10^5^ cells, respectively) were injected in the right striatum of Rnu rats. Tumor development was monitored over-time—at 7 dpi only for U87 xenografts or at 7, 25, 35, and 40 dpi for CSC xenografts. At least three animals per each time point were analyzed, euthanized immediately after the MRI exam and perfused with the vessel labeling protocol. Brains were then dissected and sliced while maintaining the same spatial orientation as the MRI scan (as in Fig. 2). The tissue slices corresponding to the region of interest studied by MRI (Fig. 3A,E,I,M) were then cleared following FRUIT100 protocol. Each slice was imaged under the confocal microscope in a 400 μm-thick z-stack, which corresponds to the maximal depth of acquisition allowed by the objective and hardware setting, or under the two-photon microscope in a 750 μm-thick z-stack to generate a high-resolution scanning of the hemisphere homolateral to the implanted xenograft. Figure 3B,F,J,N shows the maximal intensity projections (MIP) of these confocal acquisitions. Given that FRUIT100 does not modify the dimensions of the original tissue, the confocal-MIP reconstructions obtained from the cleared tissues can be accurately superimposed onto the corresponding MRI scan (Figs. 3C,G,K,O, 4A–D, 5A–D, 6A–E) allowing a very detailed side-by-side comparison between T2w or T1w + c in vivo scans and ex vivo confocal images (Fig. 3Q).

As expected, by 7dpi, U87-derived xenografts formed a tumor mass with very high cell density and clear-cut boundary (Fig. 3B,C1). The tumor boundary corresponded to a region of blood brain barrier disruption, as evidenced by using T1w and contrast agent (Fig. 3D). On the other hand, T2w imaging highlighted a hyperintense signal extending largely beyond the tumor boundary (arrowheads in Fig. 3A–C and quantification in Fig. 3R), which represents edema.

In contrast to U87-derived xenografts, there were no clear-cut boundaries for CSC-derived xenografts. Rather, a high-cell density core whose total area increased over time from 25dpi through 40dpi, could be similarly identified by both T2w and T1w + c imaging (Fig. 3E,H,I,L,M,P). However, at higher magnification, several GFP + cells were detected throughout the brain parenchyma, spreading several microns away from the high cell-density xenograft core. This phenomenon was evident at 25dpi (Fig. 3G1) and became more pronounced at later time-points (Fig. 3K1,O1).

Cells from CSC-derived xenografts were found in the cortex and corpus callosum (arrowheads in Fig. 4D and in Figs. 5D,B1 and 6A,E1–2), which suggests a high propensity to travel far away from the original injection site (asterisks in Figs. 4A, 5A and 6A,E). The quantification of total area of GFP signal confirmed a higher spreading of GFP + cells beyond the boundaries of the lesions that were highlighted by MRI (Fig. 3R). Interestingly, at all time-points of analysis we detected many GFP + CSC-derived cells closely associated with the neurovasculature in brain regions surrounding the high-cell-density tumor core (arrows in Fig. 3G1,K1,O1 and in Fig. 5B2). This association was further confirmed by analysis of the cross-section of the z-stacks obtained by confocal microscopy (400 μm thickness, arrows in Fig. 4E and corresponding 3D reconstruction in Fig. 4F) or through 2-photon (2P) microscopy (750 μm thickness, arrows in Fig. 5E and corresponding 3D reconstruction in Fig. 5F, arrows in Fig. 6F). 2P microscopy was used in this study to demonstrate the ability to acquire throughout the full thickness of the slice cleared with FRUIT 100.

## Discussion

In this work we developed an approach to correlate in vivo MRI images and ex vivo high-resolution fluorescence images of brain neurovasculature, and of the anatomical distribution of cancer cells/cancer stem cells. To this aim we: (i) utilized the Rnu rat model and validated critical differences in cellularity and invasiveness of xenografts derived from the commonly used U87 human GBM cell line and xenografts produced by patient-derived cancer stem cells^12,24,28^; (ii) developed an approach for slicing rat brain tissue into sections with the same thickness and spatial orientation as adopted for in vivo MRI imaging in order to correlate in vivo analyses and ex-vivo imaging via scanning confocal microscope; (iii) improved a clearing protocol compatible with lipophilic dyes to obtain high tissue transparency and simultaneously preserve tissue morphology, with no significant shrinkage or expansion^26^; (iv) demonstrated that the technique, hereby described, could efficiently be exploited at the preclinical level to investigate the extent of correlation between the distribution of cancer cells throughout the brain parenchyma and critical anatomical and physiological alterations—like displacement of healthy brain tissue, induction of inflammation, and breakdown of the BBB—as evidenced by MRI.

Recent advancement in tissue clearing techniques and fluorescent reporter cell tracing tools^19,21,29^ have facilitated the exploration of whole tissue architecture with unprecedented detail. Applied especially to mouse and rat brains, these advancements have led to new insights into brain anatomy, including the neurovasculature, spine density, axonal and dendritic networks^30,31^ and cellular connectivity in health and disease^21,32,33^. For this kind of applications, light-sheet (LS) fluorescence or 2P microscopy are often exploited^29,32,34^. These approaches facilitate the exploration of whole cleared brain with high resolution while overcoming the technical limitations of standard laser scanning confocal microscopes.

Moreover, combining ex vivo high-resolution fluorescence microscopy with in vivo non-invasive imaging approaches, such as MRI, would greatly improve the clinical translation of many preclinical observations^35^. This is particularly relevant for those pathologies where clinical MRI protocols are already extensively applied but further improvements could be implemented based on ex-vivo high-resolution imaging. Brain tumors, especially glioblastoma multiforme (GBM), fit this description. In fact, many endeavors have been made to try to optimize MRI protocols in a way that better stratifies patients^9,21,24^. Furthermore GBM therapy and follow-up procedures would greatly benefit from the development of new non-invasive imaging approaches capable of: (i) tracking surviving cells that spread throughout the parenchyma into regions far away from the primary lesion after surgical resection or chemotherapy; (ii) discriminating between the tumor mass and tissue damage like edema or inflammation; (iii) monitoring treatment response, especially in the context of pseudo-progression and radiation necrosis after irradiation, or pseudoresponse in the context of immunotherapy^36^.

The primary goal of this work was to create the capability to reproduce ex-vivo the same orientation, sampling (choice of the region of interest and thickness of slices) and dimensions of the tissue analyzed during MRI in vivo. Many tissue clearing protocols have been described that vary in efficiency in their ability to improve transparency of thick tissues while simultaneously preserving the detectability of critical reporters such as fluorescent proteins or dyes. The rat brain is particularly challenging, given the larger dimensions of the tissue as compared to the mouse brain, for which many whole tissue-clearing protocols have been validated. Also, the rat brain has a high lipid content due to myelin which hinders transparency whenever passive detergent-free protocols are used. Moreover, despite the fact that protocols for whole rat brain clearing have already been described^32^, the equipment and time required for acquisition of a full rat brain using these protocols could limit their widespread applicability at many laboratories that lack the proper instrumentation. In this work we chose the FRUIT tissue clearing protocol because it allows preservation of both fluorescent reporter proteins and lipophilic dyes. Preservation of the latter is critical for applications such as detection of the neurovasculature with vessel labeling by means of infusion of the lipophilic dye DiI^37^. By adopting a tissue sectioning approach which reproduces the spatial orientation and sampling of MRI slicing, we can overcome the above-mentioned hurdles by producing tissue sections of a thickness (0.75 mm) that allows for the application of a very efficient clearing protocol consisting only of a 24 h incubation step in a 100% fructose plus urea solution without significantly altering tissue dimensions as compared to the pre-clearing measurements. This allowed imaging of the cleared tissue by use of: (i) a standard scanning confocal microscope equipped with a 20 × dry objective and a motorized stage that can acquire a large region of the tissue up to a depth of about 400 μm; as well as (ii) a 2P microscope, that allows imaging throughout the full tissue thickness.

This approach showed that U87-derived xenografts remain localized at the injection site and did not diffuse beyond the tumor boundary. However, the rapidly proliferating tumor cells give rise to a tumor mass that causes extensive tissue damage, as evidenced by the large area characterized by hyperintense signal in T2w imaging protocols that extends beyond the tumor boundaries. Based on the merged T2w + confocal image, we can identify a region of hyperintense signal surrounding the tumor mass that is likely due to tumor-induced edema or inflammation. In contrast, we highlighted that the CSC-derived xenografts display a prominent tendency of cancer cells to diffuse extensively throughout the brain parenchyma, beyond the injection site. Strikingly, the presence of cancer cells in these areas did not result in prominent changes in the T2w and T1w + c signals, contrary to what was observed with U87-derived xenografts. This is likely due to the ability of CSCs to infiltrate the brain parenchyma as single cells.

In our study, we manually aligned the in vivo MRI and ex-vivo confocal images, based on visual assessment of some anatomical regions distinguishable with both imaging modalities (such as the lateral ventricles and choroid plexus, corpus callosum and hippocampus). Even if this approach is not perfect and could be susceptible to user bias, we managed to reach very good overlap. Currently, we are working on developing computational tools to aid user-independent co-registration and at the same time the quantification of spatial overlap between reference anatomical regions identified with MRI or confocal imaging^38^.

Overall, our technique could be exploited to foster the validation of novel MRI approaches aiming at identifying tumor infiltration beyond macroscopic margins and quantifying tumor neoangiogenesis. For instance, computational methods allowing unbiased discrimination of the lesion^10,39^ could be easily validated with our protocol, thanks to the possibility to track precisely the GFP-expressing cancer cells in high-resolution confocal images co-registered with MRI.

On the other hand, functional imaging allows in-depth investigation of tumor-dependent changes in perfusion of specific brain areas, providing information on blood volume, blood flow and mean transit time (with dynamic susceptibility contrast imaging, DSC-MRI) and perfusion, capillary permeability and surface area (via dynamic contrast enhanced imaging, DCE-MRI)^12,21,24^. Additionally, diffusion MRI protocols such as diffusion tensor imaging (DTI) or neurite orientation dispersion and density imaging (NODDI)^13^ allow the investigation of microstructural alterations (mainly white matter damage) and discrimination of CSF, extracellular and intracellular water. The results obtained with perfusion (DSC-MRI), permeability (DCE-MRI) or diffusion (DTI and NODDI) protocols could be accurately validated with our technique, by means of the co-registration of MRI with high-resolution confocal images obtained upon in vivo labeling of vessels (already explored in this work)^40^.

## Conclusion

We developed a method for slicing rat brain tissue into sections of the same thickness and spatial orientation adopted for in vivo MRI and improved an existing tissue-clearing protocol (FRUIT) to obtain high tissue transparency with no significant tissue shrinkage or expansion. We envision that the application of a simplified but at the same time very reliable tissue-processing and clearing method, like the one described in this paper, that is capable of preserving fluorescent reporters/dyes and tissue morphology while facilitating easy correlation between in vivo MRI and ex-vivo high-resolution fluorescence imaging microscopy, may become a valuable tool to aid in the validation of new in vivo MRI protocols for in-depth analysis and monitoring of GBM evolution and response to treatment.

## Material and methods

### Cell cultures

The U87-MG human glioblastoma cell line was purchased and kindly donated by Dr Rossella Galli, San Raffaele Scientific Institute. The cancer stem cell (CSC) line nr. 1022 was originally isolated through the neurosphere assay^28^ from a primary human GBM tissue specimen and kindly donated by Dr Rossella Galli, San Raffaele Scientific Institute. U87-MG and CSC cell lines were transduced with a lentiviral vector coding for the green fluorescent protein (GFP) under the hPGK constitutive promoter, as previously reported^25^. GFP expression was verified by flow cytometry, confirming high and homogeneous levels of the reporter gene both in U87 and CSC cell cultures. U87-MG cells were cultured at 37 °C, 5% CO_2_ with Dulbecco’s Modified Eagle Medium (DMEM) supplemented with 10% heat-inactivated fetal bovine serum (FBS), 4 mM glutamine and 1% penicillin/streptomycin. The medium was changed two or three times a week. CSCs were maintained at 37 °C, 5% CO_2_ in Human Neurocult Basal (STEMCELL Technologies) supplemented with Hormone Mix Proliferation (STEMCELL Technologies), 1% penicillin/streptomycin, 2 μg/ml Heparin, 20 ng/ml recombinant human EGF and 10 ng/ml recombinant human bFGF2.

### In vivo studies

#### Immunocompromised rats

Crl:NIH-Foxn1rnu rats (here named RNU) were purchased from Charles River. All the experiments and the protocol proposed in the project were examined and approved first by the Institutional Animal Care and Use Committee (IACUC) and then authorized by the Italian Ministry of Health. Procedures involving animals and their care were conducted according to the institutional guidelines that are in compliance with national and international laws and policies. The rats were maintained in a SPF environment with access to food and water ad libitum.

#### Brain glioblastoma model

Two months old RNU rats (n ≥ 4 animals per time-point injected with either U87 or CSCs) were anesthetized intraperitoneally with a solution of ketamine and xylazine (150 mg/kg Ketamine/10 mg/kg xylazine) and positioned in a stereotactic head frame. Injections were performed in the striatum using the following coordinates (relative to bregma): AP + 0.5 mm; ML -3.5 mm; depth − 5.5 mm, mouth bar − 3.3 mm. A Hamilton syringe (26 S Gauge) was used to inject 5 μl of cells (2.5 × 10^5^ U87 cells or 5 × 10^5^ CSCs), at flow rate of 0.5 μl/min. After injection, the needle was left in place for an additional 1 min and then slowly withdrawn. After recovery, animals were monitored three times a week for functional alterations and changes in body weight.

#### Injection of SPIONs in the brain parenchyma

Two 10-weeks old Wistar rats were anesthetized intraperitoneally with a solution of ketamine and xylazine (150 mg/kg Ketamine/10 mg/kg xylazine) and positioned in a stereotactic head frame. Injections were performed in the corpus callosum or in the striatum using the following coordinates relative to bregma, respectively: AP − 3.3 mm; ML ± 2.5 mm; depth − 2.5 mm, mouth bar − 3.3 mm; AP + 0.5 mm; ML ± 3.5 mm; depth − 5.5 mm, mouth bar − 3.3 mm. A Hamilton syringe (26 S Gauge) was used to inject 2 μl of super-paramagnetic iron oxide polycaprolactone nanoparticles (10 mg/ml iron content) at flow rate of 0.5 μl/min. After injection, the needle was left in place for an additional 1 min and then slowly withdrawn. After recovery, the distribution of SPIONs was first assessed in vivo by MRI and then ex-vivo by histology on PFA-fixed brain slices as described in “Tissue-processing, histology and immunohistochemistry” below.

#### In vivo magnetic resonance imaging (MRI)

Brain MRI acquisitions were performed 3 days post administration for SPION-injected rats, 7 days post transplantation for U87-cells injected rats, and at three different time points (25, 35 and 40 days post injections) for animals transplanted with CSCs. At each time-point, rats were anesthetized by isoflurane (4% in 100% O_2_ for induction, and 2% in 100% O_2_ for maintenance) and brain acquisitions were performed on a small animal–dedicated 7 T scanner (30/70 BioSpec; Bruker, Ettlingen, Germany) equipped with a mouse-head dedicated four-channel phased-array coil. The animals were positioned prone on the dedicated heated apparatus. The image acquisition was performed on coronal plane. For U87- or CSC-xenotransplanted rats, a Rapid Acquisition with Refocused Echoes (RARE) T2-weighted sequence (time of repetition: 3007 ms, echo time: 36 ms, voxel size: 90 × 90 × 750 μm) and two T1-weighted sequences (time of repetition: 526 ms, echo time: 8 ms, voxel size: 90 × 90 × 750 μm) were acquired before and after infusion of gadolinium contrast medium (Gadobutrole, Gadovist). First, 100 μL of contrast was diluted in 900 μL of physiological solution. The diluted solution was then injected in the tail vain using an infusion pump (MRI compatible syringe pump, Harvard Apparatus, Holliston, MA, USA). The total injected volume was 0.08 mL, at a rate of 600 μL/s. At least 4 animals per time-point (injected with either U87 or CSCs) were analyzed. For SPIONs-injected rats, a RARE T2-weighted sequence (time of repetition: 4000 ms, echo time: 36 ms, voxel size: 90 × 90 × 750 μm) was acquired.

### Ex vivo studies

#### Vessel labeling

Tumor bearing rats (n ≥ 4 animals per time-point injected with either U87 cells or CSCs) were deeply anesthetized with a solution of ketamine and xylazine (150 mg/kg Ketamine/10 mg/kg xylazine) and then perfused using a peristaltic pump. Animals were perfused first with 20 mL of PBS 1X, then with 40 mL of DiI solution (0,1 mg/mL), and finally with 40 mL of 4% paraformaldehyde (PFA) in PBS. DiI solution was prepared fresh immediately before the euthanasia, as described in^37^. Briefly: (i) DiI (1,1′-dioctadecyl-3,3,3′,3′-tetramethylindocarbocyanine perchlorate) was first dissolved in 100% ethanol at the concentration of 1 mg/ml; (ii) DiI-diluent was prepared by mixing three parts of PBS 1X and one part of Glucose 10% in water. The vessels were stained by intracardiac perfusion with 40 mL of PBS 0.1 M pH 7.4, followed by 20 mL of DiI diluted at a final concentration of 0.02 mg/ml in DiI-diluent and then finally by 20 mL of PBS 0.1 M pH 7.4 to wash the dye in excess. After perfusion, the brain was carefully dissected and post-fixed in phosphate-buffered 4% paraformaldehyde (PFA) for 5 days. Tissues used for histology were obtained by standard perfusion with PBS 0.1 M pH 7.4 followed by 10% formalin-Zn as previously described.

#### Tissue-processing, histology and immunohistochemistry

Brains were cut using a vibratome using the same thickness set and same spatial orientation applied for MRI (750 μm) image acquisition. To do so, brains were included in 3% agarose in PBS and positioned relative to the cutting blade with the same orientation assumed by the brain in the MRI scanner, so that the slices of brain could reproduce the sampling applied during MRI acquisition.

For the histology, 750 μm-thick brain slices were placed in embedding cassettes and processed for paraffin inclusion. Then, tissue slices (5 μm-thick) were cut with a microtome, collected on glass slides and subsequently deparaffinated, stained with hematoxylin and eosin and mounted in DPX with glass coverslips as previously described^25^. The Aperio ScanScope XT digital slide scanner (Leica Biosystems) was used to image the entire stained slide at 20 × magnification to create a single high-resolution digital image.

For the immunohistochemistry, 750 μm-thick brain slices were dipped for three days in sucrose 30% in PBS pH 7.4, then embedded in Optimal Tissue Compound (TissueTek) and stored at − 80 °C until the experiment. Samples were cut in 20 μm sagittal sections at the cryostat and collected on glass slides. Sections were then stained as previously described^41^ using the following primary antibodies: mouse anti-human Nuclei (Millipore, 1:100 dilution) and rabbit anti-Ki67 (Leica, 1:100 dilution). After the staining, slices were extensively washed in PBS and then counterstained for nuclei (with DAPI at 0.5 μg/ml) for 10 min. For detection of SPIONs, Prussian Blue staining was performed as previously described^42^ to highlight iron and was followed by incubation for 10 min at room temperature in Nuclear Fast Red solution (Sigma-Aldrich) to highlight the anatomical structures.

#### Tissue-clearing procedure

Clearing solutions were prepared as previously described^26^. Briefly, fructose solutions were prepared in water at the concentration of interest and then used to dissolve urea. Immediately before use, α-thioglycerol was added to the solution. The FRUIT clearing protocol consisted of the following incubations: 8 h each in 35, 40, and 60% (wt/vol) FRUIT, 12 h in 80% (wt/vol) FRUIT and 24 h in 100% (wt/vol) FRUIT. All incubations were performed at 37 °C. After clearing, samples were stored in 100% (wt/vol) FRUIT at 4 °C up to the time of acquisition.

#### Image acquisition and analysis

To evaluate changes in tissue dimensions and morphology that may have occurred upon application of the clearing protocol, the area and horizontal and vertical diameters of each tissue slice were measured before and after clearing. Briefly, the tissue slice was laid down on a paper sheet with a printed grid and then imaged at brightfield with a camera mounted on a GelDoc (Bio-rad) apparatus. Section area and diameters were measured on the digitized images with Fiji public domain software^43^.

To acquire high-resolution fluorescent microscope images of the cleared tissues, each cleared tissue slice was dipped in FRUIT 100 solution and positioned within a plastic cryomold sealed on a glass coverslip. Then, the coverslip was mounted on a motorized-stage and acquired at 20 × with a SP8 Leica scanning confocal microscope. A 400 μm-thick z-stack (0.5 μm stem-size) was acquired in the FITC and TRITC channel to visualize the GFP-labeled cancer cells and DiI-labeled vessels, respectively. To cover the region of interest, which consisted of either the brain hemi-section containing the GFP-labeled xenograft or the full slice, a stitched image was acquired.

Two-photons images were obtained using a Nikon A1-MP + Two-photon Microscope (from Nikon, Japan) with Coherent NIR Camaleon Ultra II laser (from Coherent, Santa Clara, USA). Images were collected with an Apo LWD 25 X (NA 1.1) water immersion objective. Tissue slices were acquired every 5 μm step-size. The excitation wavelength NIR laser was 900 nm for GFP + cancer cells and 700 nm for DiI-stained vessels.

To quantify and compare the area occupied by GFP + cells with the extent of hyperintense signal highlighted with MRI (T2w or T1w + c), the confocal image was co-registered on the corresponding digital slice obtained by MRI using GIMP (GNU Image Manipulation Program v.2.10.8; https://gimp.org). Image segmentation and measurement of the area occupied by GFP + cells or of the hyperintense signal highlighted with T2w or T1w + c was performed through Fiji public domain software v.1.52p; https://imagej.net/Fiji^43^. The pipeline developed for image segmentation and analysis is available on demand.

## Supplementary information

Supplementary Information.

Supplementary Figure S1.

Supplementary Figure S2.

## Data Availability

The raw data generated and analyzed for this manuscript and the image analysis pipelines developed in this work are available via request from the authors, upon signature of a formal data sharing agreement.

## Abbreviations

GBM: Glioblastoma
MRI: Magnetic resonance imaging
T2w: T2-weighted
T1w + c: Post-contrast T1-weighted
CSC: Cancer stem cells
ROI: Region of interest
