# Supplementary material for: Lipophilic dye-compatible brain clearing technique allowing correlative magnetic resonance/high-resolution fluorescence imaging in rat models of glioblastoma
Source: Sci Rep. 2020 Oct 21;10:17974. doi: 10.1038/s41598-020-75137-y (PMC7578790; doi:10.1038/s41598-020-75137-y)
Supplement: Supplementary file 1 — Supplementary Information. [file 41598_2020_75137_MOESM1_ESM.docx]

Lipophilic dye-compatible brain clearing technique allowing correlative Magnetic Resonance / high-resolution fluorescence imaging in rat models of glioblastoma.

Marco Peviani, Giorgia Spano, Antonella Pagani, Gianluca Brugnara, Cesare Covino, Rossella Galli, Alessandra Biffi, Letterio S. Politi

**Supplementary material**

**Supplementary figure 1**

**Supplementary figure 2**

**Supplementary figure S1. Human nuclei staining and Ki67 proliferation marker in U87 and CSC-xenografts.**

Representative laser scanning confocal microphotographs of human nuclei (green signal), Ki67 porliferation marker (red signal) and DAPI nuclear stain (blue) on U87 or CSC-tumor bearing representative rats. Scale bar = 25 μm.

**Supplementary figure S2. Validation of the tissue slicing and vessel labeling protocols.**

Wistar rats were injected with SPIONs (10 mg/ml iron) monolaterally into the corpus callosum or into the striatum. Qualitative comparison among T2-weighted MR images (in A, D), ex-vivo brain slices obtained as described in Fig. 2 (in B, E) and 20 μm-thick cryostat sections stained with Prussian Blue and Nuclear-Fast Red (in C, F), highlights a good correlation between ex-vivo tissue samples and in vivo MRI. Arrowheads in A-F highlight the needle trace at the injection site. The hypo-intense signal in A, D corresponds to SPIONs localized in the needle trace as evidenced by Prussian Blue stain (arrowheads in C, F). Arrows in D highlight a hyper-intense signal consistent with inflammation/edema in the sham-injected site (arrows in E). Arrows in C, F highlight the iron from SPIONs stained with Prussian Blue. hip = hippocampus; cc = corpus callosum; LV = lateral ventricle.
