# Supplementary figures and images for: Lipophilic dye-compatible brain clearing technique allowing correlative magnetic resonance/high-resolution fluorescence imaging in rat models of glioblastoma
Source: Sci Rep. 2020 Oct 21;10:17974. doi: 10.1038/s41598-020-75137-y (PMC7578790; doi:10.1038/s41598-020-75137-y)

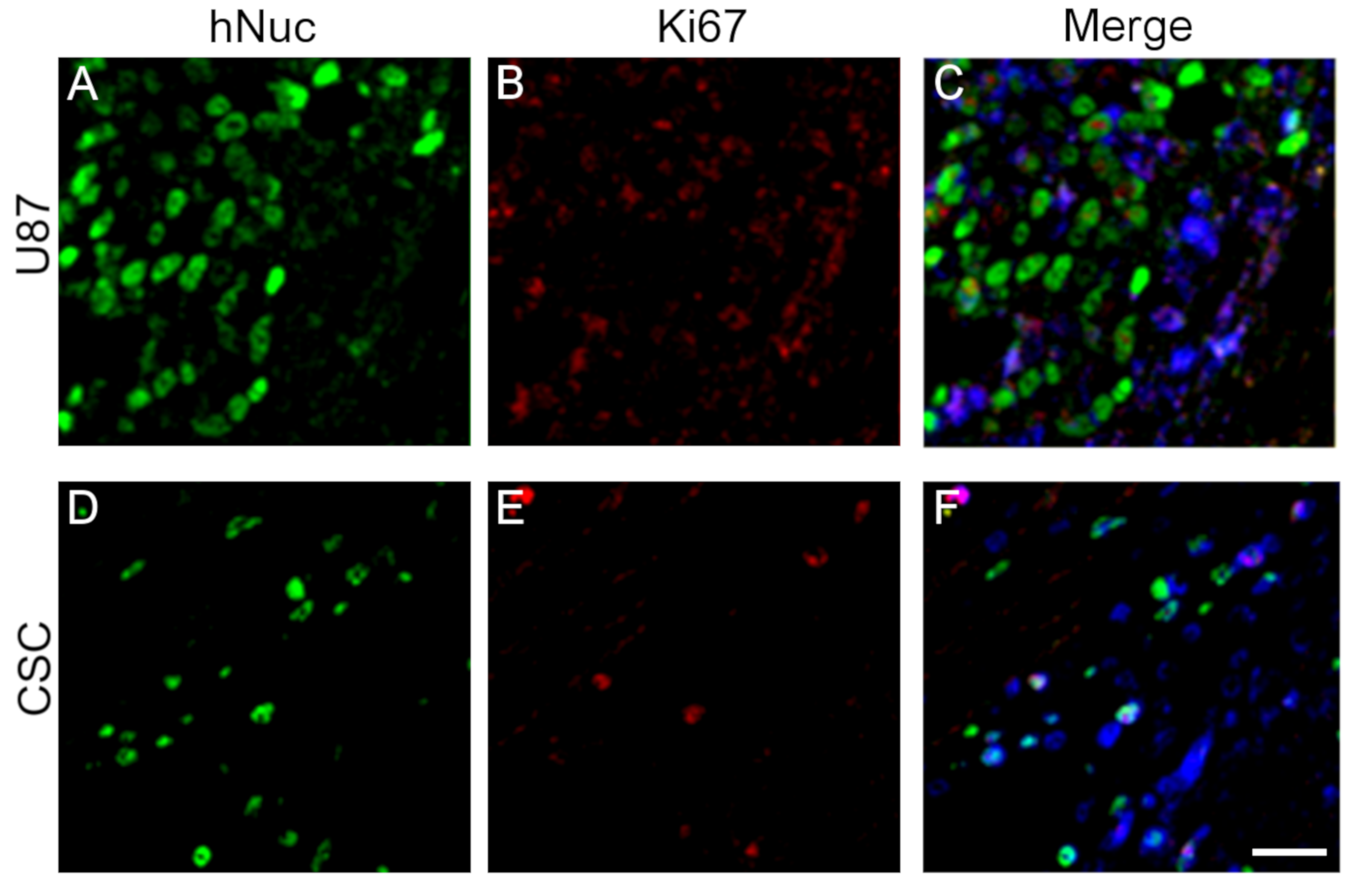

Supplement: Supplementary file 2 — Supplementary Figure S1. [file 41598_2020_75137_MOESM2_ESM.tif]

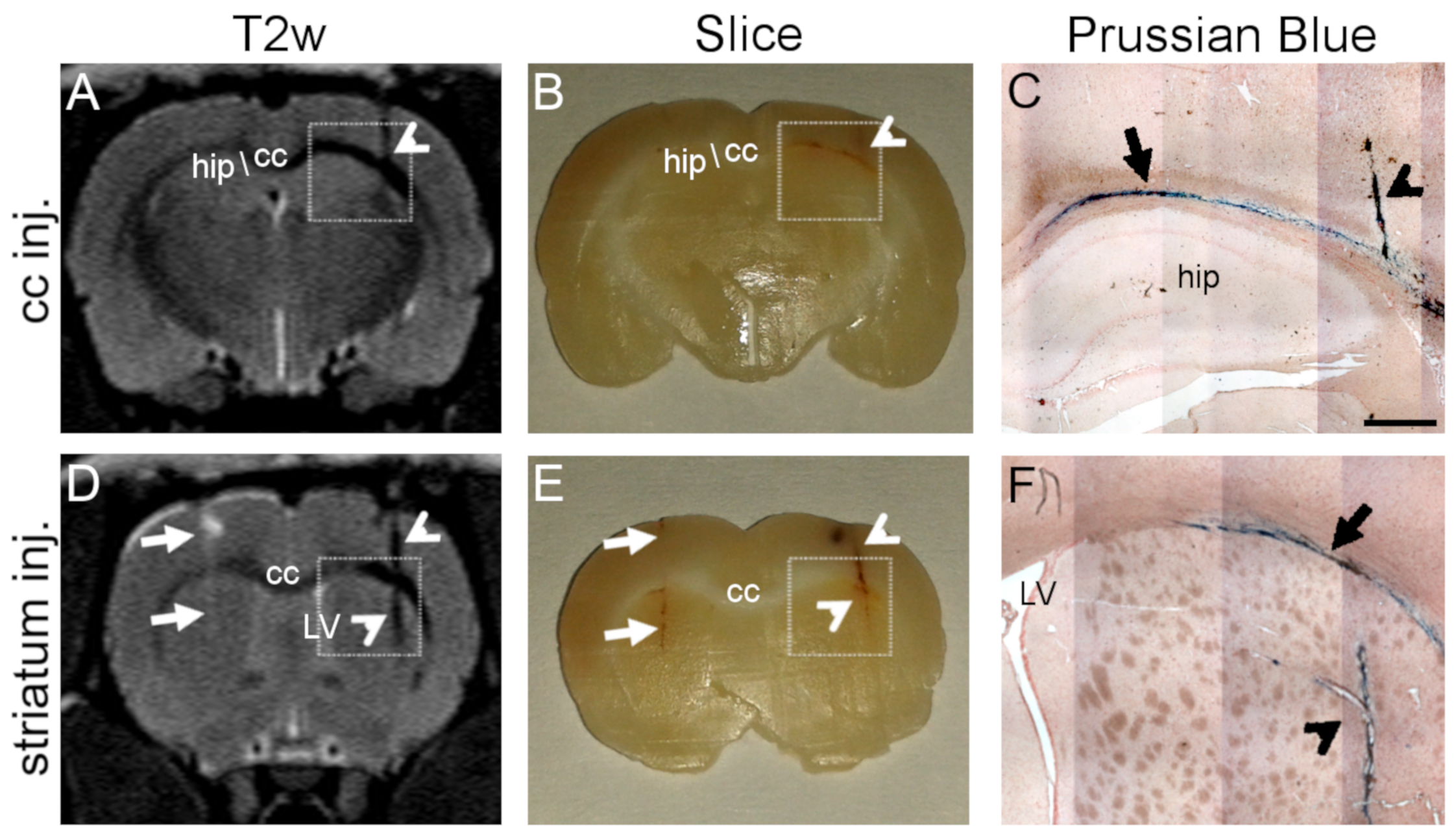

Supplement: Supplementary file 3 — Supplementary Figure S2. [file 41598_2020_75137_MOESM3_ESM.tif]
